# Digitale, personalisierte Gesundheitsinformationsangebote von Ärzt*innen: Ergebnisse einer Befragung von Patient*innen und Ärzt*innen zu Akzeptanz und Anforderungen

**DOI:** 10.1007/s00103-023-03750-z

**Published:** 2023-08-15

**Authors:** Elena Link, Paula Memenga

**Affiliations:** 1 Institut für Publizistik, Johannes Gutenberg-Universität Mainz, Jakob-Welder-Weg 12, 55128 Mainz, Deutschland; 2 Institut für Journalistik und Kommunikationsforschung, Hochschule für Musik, Theater und Medien Hannover, Hannover, Deutschland

**Keywords:** Informationsangebote, Digitale Transformation, Patient*innen, Ärzt*innen, Nutzungsintention, Information services, Digital transformation, Patients, Physicians, Intention to use

## Abstract

**Hintergrund:**

Die Aneignung von Gesundheitsinformationen ist grundlegend für die Verantwortungsübernahme von Patient*innen. Um dies zu unterstützen, müssen neue Wege der Informationsbereitstellung gefunden werden. Im vorliegenden Beitrag werden die digitale, personalisierte Bereitstellung von Gesundheitsinformationen durch Ärzt*innen betrachtet und ihre Verbreitung sowie die Nutzungsintention und Anforderungen von Patient*innen und Ärzt*innen untersucht.

**Methoden:**

Durchgeführt wurden jeweils eine Online-Befragung einer für die deutsche Bevölkerung stratifizierten Stichprobe von Patient*innen (*N* = 1000) sowie eine Online-Befragung von Ärzt*innen (*N* = 364) zum Thema digitale, personalisierte Gesundheitsinformationsangebote. Es wurden die Art der Informationsbereitstellung im ärztlichen Setting, die Nutzungsintention sowie Anforderungen an digitale Informationsangebote erfasst.

**Ergebnisse:**

Digitale, personalisierte Informationsangebote sind bisher selten. Patient*innen würden ein solches Angebot allerdings befürworten, während Ärzt*innen skeptischer eingestellt sind. Die Patient*innen legen Wert auf die Nutzerfreundlichkeit und die Informationsqualität. In Bezug auf die Darstellungsform werden Texte tendenziell wichtiger eingeschätzt als Videos. Ärzt*innen ist dagegen wichtig, dass das Angebot von einem oder einer vertrauenswürdigen Anbieter*in stammt, leitlinienkonform und kostenlos ist.

**Diskussion:**

Die Potenziale der digitalen Transformation der Informationsbereitstellung werden bisher nur bedingt ausgeschöpft. Während bei Patient*innen die Grundlage einer erfolgreichen Implementierung gegeben zu sein scheint, gilt es, aufseiten der Ärzt*innen Vorbehalte abzubauen, den Nutzen entsprechender Angebote zu kommunizieren und systemische Anreize zu schaffen.

## Abstract (en)

**Background:**

The acquisition of health information is fundamental for patient empowerment. In order to support this, new ways of providing information must be found. In this article, the digital personalized provision of health information by physicians as well as the intended use and requirements of patients and physicians are examined.

**Methods:**

An online survey of a sample of patients stratified for the German population (*N* = 1,000) and an online survey of physicians (*N* = 364) on the subject of digital personalized health information services were conducted. The type of information provision in the medical setting, the intention to use it, and the requirements for digital information services were assessed.

**Results:**

Digital personalized information services are still rare. However, patients would support such a service, while physicians are more skeptical. Patients value usability and information quality. With regard to the form of presentation, texts tend to be rated more important than videos. For physicians, on the other hand, it is important that the service comes from a trustworthy provider, complies with guidelines, and is free of charge.

**Discussion:**

The potential of the digital transformation of information provision has so far only been utilized to a limited extent. While the basis for successful implementation seems to be given among patients, physicians still need to overcome their reservations, the benefits of such services need to be communicated, and systemic incentives must be created.

## Hintergrund

Ein angemessener Umgang mit Gesundheitsinformationen ist grundlegend für die Übernahme von Verantwortung für die eigene Gesundheit, den Umgang mit gesundheitsbezogenen Unsicherheiten und die Beteiligung an gesundheitsbezogenen Entscheidungen [1–3]. Bei der Suche, Bewertung, Aneignung und Anwendung von Gesundheitsinformationen stehen Patient*innen jedoch häufig vor Herausforderungen: Gesundheitsinformationen sind oft vielschichtig, komplex und für Laien schwer verständlich [4, 5]. Zusätzlich können insbesondere bei kontroversen Themen (z. B. Impfungen oder COVID-19) irreführende und falsche Informationen die eigene Meinungsbildung und Entscheidungsfindung erschweren [6]. Patient*innen können ebenso dadurch herausgefordert sein, dass bestimmte Quellen nur bedingt zugänglich sind. So zählen Ärzt*innen in Deutschland weiterhin zu den präferierten und besonders vertrauenswürdigen Quellen für Gesundheitsinformationen [7, 8], es fehlt ihnen jedoch oft die Zeit, ihre Patient*innen umfassend zu informieren [9, 10]. Auch wenn nicht erfüllte Informationsbedürfnisse zu den Treibern der eigenen Suche nach Gesundheitsinformationen zählen [11], sind nicht alle Bevölkerungsgruppen gewillt und befähigt, selbst passende Informationen zu finden und anzuwenden [12]. So scheinen trotz neuer digitaler Möglichkeiten für mehr Chancengleichheit soziale Ungleichheiten fortzubestehen [13].

Um Patient*innen bei der Wissensaneignung zu unterstützen und ihre Verantwortungsübernahme zu fördern, gilt es zu hinterfragen, wie neue Wege der Informationsbereitstellung im Gesundheitssystem aussehen könnten. Da die Kommunikation und Vermittlung von Gesundheitsinformationen wesentliche Bestandteile der psychosozialen Dimension der Gesundheitsversorgung sind [14], werden in diesem Beitrag Ärzt*innen in ihrer Rolle als vertrauenswürdige Mittler*innen fokussiert [8]. Es wird postuliert, dass zukünftig ein zusätzliches digitales Informationsangebot von Ärzt*innen einen möglichen Weg der personalisierten und problemorientierten Informationsbereitstellung darstellt. Es könnte sich dabei beispielsweise um eine webbasierte Plattform handeln, über die Ärzt*innen ihren Patient*innen personalisierte, d. h. auf die individuelle Krankheitssituation zugeschnittene, evidenzbasierte Informationen (z. B. zu Symptomen, Behandlungsoptionen, Präventions- und Früherkennungsmaßnahmen) zur Verfügung stellen. Die Patient*innen könnten sich die Informationen wiederum in Ruhe zu Hause am Computer oder auf einem Smartphone ansehen. Die Informationen werden auf Basis der zuvor stattgefundenen ärztlichen Sprechstunde ausgewählt und ergänzen diese. Durch die Bereitstellung solcher digitalen, evidenzbasierten, personalisierten Informationen von Ärzt*innen erhalten Patient*innen ein qualitativ hochwertiges Informationsangebot, das sie von der eigenen Recherche und Qualitätsbewertung entlastet und sich vor allem durch den Zugang und Selektionsprozess von Gesundheitsinformationsportalen im Internet abgrenzt.

Mit dem Ziel, aus kommunikationsstrategischer Sicht Empfehlungen für eine solche Informationsbereitstellung abzuleiten, beleuchten wir in der vorliegenden Arbeit zunächst den *Status quo* der ärztlichen Informationsbereitstellung in Deutschland sowie die Akzeptanz für digitale, personalisierte Gesundheitsinformationsangebote von Patient*innen und Ärzt*innen. Bislang ist wenig zur Verbreitung und Akzeptanz entsprechender Angebote in Deutschland bekannt. Die hohe Bedeutung des Internets für die eigenständige Informationssuche von Patient*innen [12, 15] sowie Studien zur Akzeptanz von versorgungsbezogenen E‑Health-Leistungen in Deutschland [16, 17] legen jedoch nahe, dass Patient*innen prinzipiell offen für digitale Angebote sind. Deutlich wird aber auch, dass die Implementierung entsprechender E‑Health-Angebote in Deutschland noch weniger vorangeschritten ist als in anderen Ländern [18, 19], in denen diese eine moderate bis eher hohe Akzeptanz erfahren [20–23]. Erste Studien unter Ärzt*innen in Deutschland zeigen, dass diese zwar häufig digitale Angebote nutzen [24], ihre Akzeptanz von digitalen Angeboten für Patient*innen jedoch moderat bis eher gering ausfällt [25–27]. So fühlen sich viele Ärzt*innen nicht kompetent genug, ihre Patient*innen zu verfügbaren digitalen Angeboten, wie beispielsweise Apps, zu beraten [27]. Hinzu kommen systemische Barrieren wie knappe Zeitressourcen und begrenzte Förder- und Unterstützungsmöglichkeiten. Damit digitale Informationsangebote in der Gesundheitsversorgung implementiert werden können, gilt es somit in einem weiteren Untersuchungsschritt zu identifizieren, welche Erwartungen und Anforderungen sowohl Patient*innen als Informationsempfangende als auch Ärzt*innen als Informationsbereitstellende an solche Angebote stellen.

In der vorliegenden Studie werden der aktuelle Stand der Informationsbereitstellung im ärztlichen Setting sowie die Erwartungen und Anforderungen an digitale, personalisierte Gesundheitsinformationsangebote aus der Perspektive von Patient*innen und Ärztinnen exploriert. Mit Blick auf Patient*innen werden folgende Forschungsfragen (FF) untersucht:

### FF 1:

Inwiefern haben Patient*innen bereits digitale, personalisierte Informationsangebote von ihrem Arzt oder ihrer Ärztin erhalten?

### FF 2:

Inwiefern würden Patient*innen ein solches Angebot nutzen?

### FF 3:

Welche konkreten Anforderungen und Erwartungen bestehen aus Patient*innen-Perspektive an ein solches Angebot?

Analog zu diesen Schwerpunkten werden auch aus Perspektive der Ärzt*innen folgende Fragen untersucht:

### FF 4:

Welche Wege der Informationsbereitstellung nutzen Ärzt*innen aktuell?

### FF 5:

Inwiefern würden Ärzt*innen ihren Patient*innen (weiterhin) ein digitales, personalisiertes Informationsangebot bereitstellen?

### FF 6:

Welche konkreten Anforderungen und Erwartungen bestehen aus Ärzt*innen-Perspektive an ein solches Angebot?

## Methoden

### Patient*innen-Befragung

Zur Beantwortung der FF 1–3 (Patient*innen-Perspektive) wurde im Juli 2021 eine quantitative Online-Befragung einer für die deutsche Bevölkerung nach Alter, Geschlecht und Bildung stratifizierten Stichprobe durchgeführt. Das Sample (*N* = 1000) wurde mittels eines Online-Access-Panels von Bilendi & Respondi rekrutiert. Die Teilnehmenden wurden zu Beginn über die Inhalte der Befragung aufgeklärt und gaben ihre informierte Einwilligung zur Teilnahme. Ein positives Ethikvotum der Zentralen Ethikkommission der Leibniz Universität Hannover und Hochschule für Musik, Theater und Medien Hannover liegt vor.

Zur Erfassung der Erfahrung mit digitalen Angeboten und der Meinung zu einem digitalen, personalisierten Informationsangebot der eigenen Ärzt*innen wurde zunächst ein solches digitales, personalisiertes Angebot beschrieben. Dabei wurde darauf verwiesen, dass es sich um eine Plattform handeln kann, auf der Ärzt*innen personalisierte, d. h. individuell auf den oder die Patient*in und ihre jeweilige gesundheitliche Problemlage zugeschnittene Informationen zu Symptomen und medizinischen Untersuchungen bereitstellen. Die Befragten gaben anschließend an, ob sie schon einmal ein vergleichbares Angebot erhalten hatten (*Ja*/*Nein*/*weiß nicht*). Zudem wurden sie gebeten, in Anlehnung an Venkatesh et al. [28] über die Bewertung von 3 Items auf einer 7‑stufigen Likert-Skala ihre Nutzungsintention anzugeben (1 *stimme ganz und gar nicht zu* bis 7 *stimme voll und ganz zu*). Basierend auf der Reliabilitätsprüfung wurden die Items zu einem Mittelwertindex zusammengefasst (α = 0,98; *M* = 4,7; *SD* = 2,0).

Um die konkreten Anforderungen an ein entsprechendes Angebot zu ermitteln (FF 3), wurden aus der Leitlinie evidenzbasierte Gesundheitsinformationen [29] verschiedene Erwartungen und Anforderungen an Gesundheitsinformationen und ihre Vermittlung abgeleitet. Die Befragten wurden jeweils gebeten, sowohl die Bedeutung (*Wie wichtig ist Ihnen, dass das digitale Angebot …?*) von Aspekten der Benutzerfreundlichkeit und Aufbereitung (z. B. Video, Text, Bilder, Möglichkeit zur Interaktion) als auch Merkmale der Informationen (z. B. verständlich, aktuell, umfangreich) und ihres Zuschnitts (z. B. Vorteile und Chancen, Nachteile und Risiken, Entscheidungshilfen) über einzelne Items auf einer 7‑stufigen Likert-Skala (1 *überhaupt nicht wichtig* bis 7 *sehr wichtig*) zu bewerten (Tab. 1). Für die Beantwortung der FF 1–3 wurde eine deskriptive Analyse der Daten vorgenommen.

**Table 1:** 

Anforderungen	*M*	*SD*
*(1) Aspekte der Nutzerfreundlichkeit und Aufbereitung*		
Leicht und intuitiv zu bedienen	6,19	1,28
Übersichtlich gestaltet	6,19	1,28
Unterhaltsam	3,60	1,91
Enthält Videos	4,20	1,90
Enthält grafische Darstellungen (z. B. Diagramme)	4,87	1,76
Enthält Texte	5,68	1,46
Enthält Bilder (z. B. Fotos, anatomische Bilder)	5,25	1,65
Bietet Möglichkeit zur Wissensanwendung (z. B. Quiz)	3,13	1,96
Bietet Möglichkeit, Fragen an die Ärztin/den Arzt zu stellen	5,63	1,53
Bietet Möglichkeit des Austauschs mit anderen Patient*innen	3,27	1,87
*(2) Merkmale der Informationen*		
Verständlich	6,35	1,22
Aktuell	6,20	1,27
Umfangreich	5,92	1,37
Von unabhängigen Quellen	5,66	1,52
*(3) Zuschnitt der Informationen*		
Auf persönliche Bedürfnisse zugeschnitten	5,86	1,43
Stellt verschiedene Optionen dar (z. B. Behandlungsmöglichkeiten)	5,59	1,45
Stellt Vorteile und Chancen dar (z. B. von einer Behandlung)	5,59	1,46
Stellt Nachteile und Risiken dar (z. B. von einer Behandlung)	5,84	1,42
Hilft bei der Klärung persönlicher Präferenzen und Werte	5,40	1,48

### Ärzt*innen-Befragung

Zur Beantwortung der FF 4–6 (Ärzt*innen-Perspektive) wurde im Frühjahr 2022 eine quantitative Online-Befragung mit in Deutschland tätigen Ärzt*innen unterschiedlicher Fachrichtungen (u. a. Allgemeinmedizin, Kardiologie, Dermatologie, Gynäkologie) durchgeführt (*N* = 364). Die Rekrutierung erfolgte über Aufrufe, die von Ärztekammern, Verbänden und Vereinen weitergeleitet wurden. Zudem wurde auf ein spezialisiertes, kommerzielles Online-Access-Panel (Dynata) zurückgegriffen. Die Ärzt*innen wurden vor ihrer Teilnahme über die Inhalte der Befragung aufgeklärt und gaben ihre informierte Einwilligung. Auch hier liegt ein positives Ethikvotum der Zentralen Ethikkommission der Leibniz Universität Hannover und Hochschule für Musik, Theater und Medien Hannover vor.

**Figure 1:**
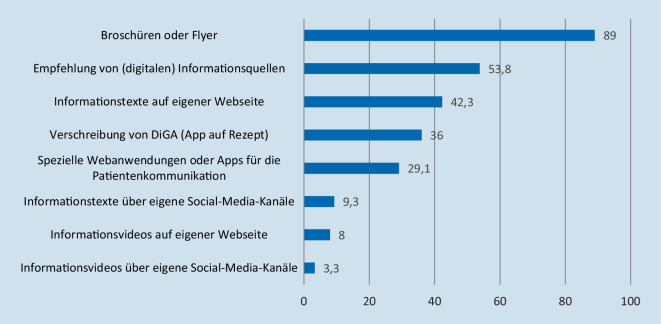

Um die genutzten Wege der Informationsbereitstellung zu identifizieren (FF 4), wurden die befragten Ärzt*innen gebeten, aus einer Liste die Informationsangebote auszuwählen, die sie ihren Patient*innen regelmäßig anbieten (Abb. 1). Zur Erhebung der Erfahrung und Meinung in Bezug auf digitale, personalisierte Informationsangebote für Patient*innen wurde den Befragten zunächst ein solches Angebot als digitale Plattform beschrieben, über die individuelle Gesundheitsinformationen (z. B. zu den Grundlagen einer Krankheit, zu Symptomen, Risiken oder Therapiemöglichkeiten) ergänzend zu den Erläuterungen in der Sprechstunde zur Verfügung gestellt werden können. Die Ärzt*innen gaben anschließend an, ob sie aktuell ein vergleichbares Angebot für ihre Patient*innen bereitstellen (*Ja*/*Nein*/*weiß nicht*) und falls ja, um welche Art von Informationen es sich dabei handelt (z. B. Informationen zu Prävention oder Behandlungsoptionen) und in welcher Form diese präsentiert werden (*Texte*/*Videos*/*Bilder*/*sonstige Formate*). Sie stuften außerdem ihre Intention ein, ein solches digitales, personalisiertes Informationsangebot weiterhin bzw. zukünftig für ihre Patient*innen bereitzustellen (FF 5). Diese Einschätzung erfolgte mittels 3 Items auf einer 5‑stufigen Likert-Skala [28] von 1 *stimme ganz und gar nicht zu* bis 5 *stimme voll und ganz zu*, die anschließend zu 2 Mittelwertindices zusammengefasst wurden. Dabei wurde unterschieden, ob die Ärzt*innen ein solches Angebot bereits nutzen (α = 0,95; *M* = 4,35; *SD* = 0,82) oder noch nicht genutzt hatten (α = 0,93; *M* = 3,27; *SD* = 1,16).

Um die konkreten Anforderungen und Erwartungen an ein entsprechendes Angebot zu ermitteln (FF 6), wurden die Ärzt*innen gefragt, die Wichtigkeit bestimmter Aspekte auf einer 5‑stufigen Likert-Skala von 1 *überhaupt nicht wichtig* bis 5 *sehr wichtig* anzugeben. Die Anforderungskategorien orientierten sich dabei an den im *Consolidated Framework for Implementation Research *[30, 31] aufgeführten Interventionscharakteristika (z. B. Quelle des Angebots, Stärke und Qualität der Evidenz, Kosten; Tab. 2). Die Ärzt*innen stuften zudem ihre Erwartungen hinsichtlich der Einfachheit der Nutzung (*M* = 3,21; *SD* = 1,01; α = 0,86) und Implementierung (*M* = 2,87; *SD* = 1,06; α = 0,90) eines solchen Angebots mittels jeweils 3 Items und in Bezug auf den persönlichen Nutzen für ihren Arbeitsalltag (*M* = 3,49; *SD* = 0,94; α = 0,91) sowie für ihre Patient*innen (*M* = 3,68; *SD* = 0,84; α = 0,91) mittels jeweils 5 Items auf einer 5‑stufigen Likert-Skala [28] von 1 *stimme ganz und gar nicht zu *bis 5 *stimme voll und ganz zu* ein. Für die Beantwortung der FF 4–6 wurde eine deskriptive Analyse der Daten vorgenommen.

**Table 2:** 

**Anforderungen**	***M***	***SD***
*(1) Quelle des Angebots*		
Wurde von Ärzt*innen entwickelt	3,81	1,02
Stammt von einem/r vertrauenswürdigen Anbieter*in	4,42	0,83
*(2) Stärke und Qualität der Evidenz*		
Es gibt wissenschaftliche Studien zur Wirksamkeit	3,90	1,00
Es gibt Erfahrungsberichte von Ärzt*innen	3,77	1,01
Es gibt Erfahrungsberichte von Patient*innen	3,64	1,05
Ist leitlinienkonform	4,23	0,97
Wird von Fachgesellschaft empfohlen	3,52	1,06
*(3) Relativer Vorteil*		
Spart in den Sprechstunden Zeit	4,15	1,00
*(4) Anpassbarkeit*		
Nach individuellen Wünschen und Vorstellungen anpassbar	4,11	0,96
*(5) Triability (Erprobbarkeit) und Unterstützung*		
Es gibt entsprechende Schulungen/Fortbildungen	3,83	1,01
Es gibt eine Service-Hotline für Probleme/Fragen	3,96	1,09
*(6) Kosten*		
Ist für Ärzt*innen kostenlos	4,28	0,92
Ist für Patient*innen kostenlos	4,05	1,07
Bereitstellung kann abgerechnet werden	3,98	1,06
**Erwartungen**	***M***	***SD***
*(1) Komplexität*		
Der Umgang mit dem Angebot ist einfach	3,21	1,01
Die Implementierung ist einfach	2,87	1,06
*(2) Persönlicher Nutzen*		
Ist nützlich im Arbeitsalltag	3,49	0,94
Ist nützlich für Patient*innen	3,68	0,84

## Ergebnisse

### Charakteristika der befragten Patient*innen und Ärzt*innen

Die Patient*innen waren im Alter von 18–74 Jahren (*M* = 51,8; *SD* = 14,4). Die Hälfte der Teilnehmenden war weiblich. 33,3 % hatten ein geringes Bildungsniveau (keinen Schulabschluss oder Hauptschulabschluss), 33,3 % hatten ein mittleres (Realschulabschluss) und 33,4 % ein hohes Bildungsniveau ((Fach‑)Abitur oder höherer Abschluss).

Die Ärzt*innen waren im Alter von 33–75 Jahren (*M* = 53,9; *SD* = 8,1) und 31,9 % waren weiblich. Insgesamt waren 97 % in einer ärztlichen Praxis und 3 % im Krankenhaus oder in anderen medizinischen Einrichtungen tätig.

### Die Perspektive der Patient*innen

FF 1 adressiert die bisherigen Erfahrungen von Patient*innen mit digitalen, personalisierten Informationsangeboten ihrer Ärztin oder ihres Arztes. Diesbezüglich gaben 3,1 % der Befragten an, schon einmal ein digitales Angebot erhalten zu haben. 2,8 % der Patient*innen konnten sich nicht erinnern und 94,1 % hatten bisher kein Angebot erhalten.

FF 2 fokussiert die Intention der Patient*innen, ein digitales, personalisiertes Informationsangebot ihrer Ärztin oder ihres Arztes zu nutzen. Die Befragten wiesen insgesamt eine mittlere bis eher hohe Nutzungsintention auf (*M* = 4,70; *SD* = 2,00). Dabei waren 58,2 % (eher) gewillt, 16,4 % unentschlossen und 25,4 % (eher) nicht gewillt, zukünftig ein solches Angebot in Anspruch zu nehmen.

Mit FF 3 sollten die Anforderungen von Patient*innen an ein digitales, personalisiertes Informationsangebot identifiziert werden (Tab. 1). Hinsichtlich der Benutzerfreundlichkeit und Aufbereitung eines solchen Angebots zeigte sich, dass eine leichte und intuitive Bedienbarkeit (*M* = 6,19; *SD* = 1,28) sowie eine übersichtliche Gestaltung (*M* = 6,19; *SD* = 1,28) besonders wichtig sind, während es nur von nachrangiger Bedeutung scheint, dass das Angebot auch unterhaltsam ist (*M* = 3,60; *SD* = 1,91) und beispielsweise ein Wissens-Quiz (*M* = 3,13; *SD* = 1,96) oder Interaktionsmöglichkeiten mit anderen Patient*innen (*M* = 3,27; *SD* = 1,87) bietet. In Bezug auf die Darstellungsform wurden Texte (*M* = 5,68; *SD* = 1,46) und Bilder (z. B. Fotos, anatomische Bilder, *M* = 5,25; *SD* = 1,65) als tendenziell wichtiger bewertet als Videos (*M* = 4,20; *SD* = 1,90) und grafische Darstellungen (z. B. Diagramme, *M* = 4,87; *SD* = 1,76). Die Befragten legten außerdem Wert auf die Verständlichkeit (*M* = 6,35; *SD* = 1,22) und Aktualität (*M* = 6,20; *SD* = 1,27) der Informationen. Ebenso wurde als eher wichtig bewertet, dass die Informationen auf persönliche Bedürfnisse zugeschnitten sind (*M* = 5,86; *SD* = 1,43), Wahlmöglichkeiten aufzeigen (*M* = 5,59; *SD* = 1,46) und sowohl Chancen (*M* = 5,59; *SD* = 1,46) als auch Risiken (*M* = 5,84; *SD* = 1,42) adressieren.

### Die Perspektive der Ärzt*innen

FF 4 fokussiert die genutzten Wege der Informationsbereitstellung von Ärzt*innen für ihre Patient*innen (Abb. 1). Der mit Abstand am häufigsten genutzte Weg (89,0 %) ist die Bereitstellung von Broschüren oder Flyern, die beispielsweise im Wartezimmer ausliegen oder in der Sprechstunde ausgehändigt werden. Etwas mehr als die Hälfte der befragten Ärzt*innen (53,8 %) gaben außerdem an, ihren Patient*innen Informationsquellen für die eigene, weiterführende Recherche (z. B. die Webseite *Gesundheitsinformation.de* oder Angebote der Bundeszentrale für gesundheitliche Aufklärung [BZgA]) zu empfehlen. 42,3 % der Befragten stellen Gesundheitsinformationen über die eigene Webseite zur Verfügung. Rund ein Drittel greift auf verschreibungspflichtige Apps (36,0 %) und speziell für die Patient*innen-Kommunikation entwickelte Webanwendungen und Apps (29,1 %) zurück. Nur wenige der befragten Ärzt*innen (9,3 %) stellen Informationstexte über Social-Media-Kanäle bereit. Selten werden Videos auf der eigenen Webseite (8,0 %) oder über Social Media (3,3 %) angeboten.

Konkret für die bisherige Nutzung und das Angebot von digitalen, personalisierten Informationsangeboten zeigte sich, dass nur ein geringer Teil der befragten Ärzt*innen (15,4 %) ihren Patient*innen ein solches Angebot anbietet. Ist dies der Fall, handelt es sich vorwiegend um informierende Texte (83,9 %). Bei 57,1 % der genutzten digitalen, personalisierten Informationsangebote sind zudem Bilder und bei 42,9 % Videos enthalten. Thematisch werden dabei insbesondere Informationen zu den Ursachen und Symptomen bestimmter Krankheiten (82,1 %), zur Prävention von Krankheiten und einer gesunden Lebensweise (73,2 %) sowie zu verschiedenen Behandlungsoptionen (73,2 %) angeboten. Rund die Hälfte der Angebote (55,4 %) enthält zudem Informationen zur Früherkennung von Krankheiten.

FF 5 fokussiert die Intention der Ärzt*innen, ihren Patient*innen ein digitales, personalisiertes Informationsangebot anzubieten. Die befragten Ärzt*innen, die ein solches Angebot bereits nutzen (15,4 %), hatten insgesamt eine hohe Intention, dieses Angebot auch weiterhin zu nutzen (*M* = 4,35; *SD* = 0,82). Dabei waren 85,7 % (eher) bereit, 12,5 % unentschlossen und 1,8 % (eher) nicht bereit, ihren Patient*innen ein solches Angebot weiterhin anzubieten. Die befragten Ärzt*innen, die bisher noch keine digitalen, personalisierten Informationen anboten (84,6 %), wiesen insgesamt eine mittlere Nutzungsintention auf (*M* = 3,27; *SD* = 1,16). Dabei waren 48,4 % (eher) bereit, 25,9 % unentschlossen und 25,6 % (eher) nicht bereit, ihren Patient*innen ein solches Angebot in Zukunft anzubieten.

FF 6 zielt darauf, die Anforderungen und Erwartungen von Ärzt*innen an ein digitales, personalisiertes Informationsangebot zu identifizieren (Tab. 2). Insgesamt bewerteten die befragten Ärzt*innen alle aufgeführten Anforderungen als (eher) wichtig. Besonders zentral scheint es, dass das Angebot von einem oder einer vertrauenswürdigen Anbieter*in stammt (*M* = 4,42; *SD* = 0,83), leitlinienkonform ist (*M* = 4,23; *SD* = 0,97) und für Ärzt*innen (*M* = 4,28; *SD* = 0,92) und Patient*innen (*M* = 4,05; *SD* = 1,07) kostenlos angeboten wird. Außerdem sollte das Angebot nach den individuellen Wünschen der Ärzt*innen anpassbar sein (*M* = 4,11; *SD* = 0,96) und Zeitersparnis in der Sprechstunde mit sich bringen (*M* = 4,15; *SD* = 1,00). Ebenso wurde Unterstützung bei der Implementierung und dem Einsatz, beispielsweise durch eine Service-Hotline (*M* = 3,96; *SD* = 1,09), gewünscht. Eine durch wissenschaftliche Studien nachgewiesene Wirksamkeit des Angebots (*M* = 3,90; *SD* = 1,00) scheint ebenfalls bedeutsam, während Erfahrungsberichte (*M* = 3,77; *SD* = 1,01) und offizielle Empfehlungen der Fachgesellschaft (*M* = 3,52; *SD* = 1,06) als etwas weniger relevant wahrgenommen wurden.

Zudem zeigte sich, dass die erwartete Komplexität eines digitalen Angebots eine Barriere darstellen könnte. Der Umgang mit dem Angebot (*M* = 3,21; *SD* = 1,01) sowie die Implementierung im Praxisalltag (*M* = 2,87; *SD* = 1,06) wurden als mittelmäßig einfach bis eher schwierig eingeschätzt. Die Erwartungen an den persönlichen Nutzen des Angebots fielen etwas positiver aus. Die befragten Ärzt*innen erwarteten sowohl für ihren Arbeitsalltag (*M* = 3,49; *SD* = 0,94) als auch für ihre Patient*innen (*M* = 3,68; *SD* = 0,84) einen eher hohen Nutzen.

## Diskussion

Wir berichten im vorliegenden Beitrag über die Befragung von Patient*innen und Ärzt*innen zum Einsatz digitaler, personalisierter Gesundheitsinformationsangebote. Damit bieten wir Einblicke zum *Status quo* der Informationsbereitstellung im ärztlichen Setting und zeigen Ansatzpunkte auf, wie digitale Angebote gestaltet werden sollten, damit sie sowohl von Patient*innen als auch von Ärzt*innen akzeptiert werden. Beides wird als Voraussetzung verstanden, um Patient*innen bei der Aneignung von Wissen über ihre eigene Gesundheit zu unterstützen und eine Grundlage für ihre Beteiligung an der Gesundheitsversorgung und gemeinsamen Entscheidungsfindung zu schaffen [2, 3]. Ärzt*innen werden im gewählten Ansatz als Mittler*innen digitaler Angebote fokussiert, weil die Kommunikation von Gesundheitsinformationen zu den psychosozialen Aspekten der Gesundheitsversorgung zählt [14] und Ärzt*innen in Deutschland ein hohes Vertrauen entgegengebracht wird [7, 8].

Die Erkenntnisse der beiden Befragungen machen in diesem Kontext zunächst deutlich, dass die Informationsbereitstellung von Ärzt*innen für ihre Patient*innen noch wenig digital erfolgt (siehe FF 1 und 4). Nur ein Bruchteil der Patient*innen berichtet, dass sie von ihren Ärzt*innen schon einmal ein digitales, personalisiertes Informationsangebot erhalten haben. Ebenso berichten die Ärzt*innen davon, dass überwiegend analoge Formen der Informationsbereitstellung wie Flyer und Broschüren eingesetzt werden. Dies steht im Einklang mit bisherigen Erkenntnissen [16, 18, 19], die zeigen, dass die Implementierung einer Vielzahl von E‑Health-Anwendungen – die nicht nur Informationsangebote, sondern auch diverse Apps zum Gesundheitsmanagement oder zur Erhebung und Dokumentation von Gesundheitsdaten einschließen – in Deutschland im internationalen Vergleich noch wenig vorangeschritten ist. Die Ergebnisse verdeutlichen zudem, dass die Informationsbereitstellung für zu Hause bei vielen Ärzt*innen schon zur gelebten Praxis zählt, wenn beispielsweise Flyer mitgegeben werden. Zusätzliche digitale Potenziale mit Blick auf einen individuelleren Zuschnitt der angebotenen Informationen und eine noch bessere Anleitung und Navigation der Patient*innen bei ihrer Wissensaneignung bleiben hingegen ungenutzt, obwohl diese nachweislich die Versorgungsqualität verbessern [32] und Patient*innen beispielsweise bei der eigenen Selektion passender Informationen entlasten.

Mit Blick auf die Entwicklungsperspektiven wurden die Nutzungsintention und die Erwartungen in den Mittelpunkt gerückt (siehe FF 2 und 5). Vonseiten der Patient*innen zeigt sich eine prinzipielle Offenheit für digitale Lösungen der Informationsbereitstellung, während Ärzt*innen zwar interessiert zu sein scheinen, aber eine tendenziell skeptischere Haltung aufweisen. Damit geht einher, dass Ärzt*innen stärker Probleme in der Implementierung entsprechender Systeme antizipieren und somit auch technische Kompetenzen als Barriere der Digitalisierung der Informationsbereitstellung deutlich werden [18, 27]. Vertiefende Analysen, die über den Fokus dieses Beitrags hinausgehen, ergaben, dass die Innovationsfreude der befragten Ärzt*innen entscheidend für die Akzeptanz eines solchen digitalen, personalisierten Informationsangebots ist, während das Alter keine Rolle spielt. Der Nutzen entsprechender Angebote für den Arbeitsalltag und die Patient*innen wird nur als mittelmäßig bis eher hoch eingeschätzt. Auch diese Haltung kann zusätzlich zur erwarteten Komplexität der Implementierung und geringen Innovationsfreude als Barriere der Digitalisierung fungieren.

Hinsichtlich der Gestaltung von digitalen Informationsangeboten (siehe FF 3 und 6) kann aus beiden Befragungen abgeleitet werden, dass aus Sicht von Patient*innen Aspekte der Nutzerfreundlichkeit sowie Informationsgehalt und -zuschnitt im Vordergrund stehen. Sie legen besonderen Wert darauf, dass entsprechende Angebote intuitiv bedienbar und übersichtlich gestaltet sind. Zudem wird Text mehr geschätzt als aufwändigere Formen der multimedialen Aufbereitung. Die bereitgestellten Inhalte sollten außerdem verständlich, aktuell und möglichst umfangreich sein sowie auf unabhängige Quellen referieren. Der Umfang schließt dabei auch ein, dass die personalisierten Informationen Vor- und Nachteile sowie verschiedene Optionen darstellen sollten. Analog zur Bedeutung unabhängiger Quellen für Patient*innen legen Ärzt*innen besonders Wert darauf, dass ein digitales Angebot von einem oder einer vertrauenswürdigen Anbieter*in stammt sowie dass der Einsatz leitlinienkonform ist. Ebenfalls wichtig ist aber auch der Kostenaspekt. So scheint es bedeutsam, dass durch das Angebot keine weiteren Kosten entstehen. Aus Sicht der Ärzt*innen sind somit externe, formale Aspekte besonders zentral. Einen bedeutsamen Grund der Zuwendung zu entsprechenden Angeboten kann es laut der Daten darstellen, dass in der Sprechstunde Zeit gespart wird. Der stärkere Einsatz digitaler Angebote könnte somit zu einer besseren Ausschöpfung begrenzter Zeitressourcen [10] beitragen.

### Limitationen und zukünftige Ansatzpunkte

Für die Interpretation der vorgestellten Erkenntnisse sollten die folgenden Limitationen der Studien einbezogen werden. Diese bieten Ansatzpunkte für zukünftige Forschung. Beide Befragungen thematisierten nur den spezifischen Ansatz der Digitalisierung der Informationsbereitstellung, der Ärzt*innen als Informationsvermittler*innen fokussiert. Demnach können keine Ansatzpunkte der Digitalisierung außerhalb des ärztlichen Settings abgeleitet werden, wenn auch die Anforderungen der Patient*innen sicherlich auf Angebote anderer Anbieter*innen übertragbar sind. Zweitens beruht die vorliegende Studie auf einer stratifizierten Stichprobe der deutschen Bevölkerung und behandelt daher diese Allgemeinheit als potenzielle Patient*innen. Zukünftige Studien sollten unterschiedliche Patient*innengruppen noch stärker differenzieren und deren konkrete Bedarfe, Erfahrungen, Erwartungen und Anforderungen spezifischer abbilden. Drittens handelt es sich ausschließlich um die Abfrage der Nutzungsintention und nicht der tatsächlichen Nutzung. Eine Vielzahl von systemischen Barrieren (z. B. limitierte Verfügbarkeit entsprechender Angebote, begrenzte Zeitressourcen für die Bereitstellung) und persönlichen Barrieren (z. B. geringe digitale Gesundheitskompetenz, geringe Innovationsfreude) kann trotz hoher Intention letztlich eine Nutzung erschweren oder verhindern. Entsprechende Bedingungen sollten ebenfalls Gegenstand zukünftiger Forschung sein. Viertens muss das Sample der Ärzt*innen-Befragung kritisch betrachtet werden. Es handelt sich hierbei um kein repräsentatives Sample und es ist davon auszugehen, dass im Sinne der Selbstselektion vor allem Ärzt*innen an dieser Befragung teilgenommen haben, die der Digitalisierung offener gegenüberstehen. Zuletzt wird in diesem Ansatz Ärzt*innen eine Rolle zugeschrieben, die i. d. R. nicht Teil ihrer Ausbildung ist und deren Ausübung insbesondere bei weniger digital affinen Ärzt*innen Unterstützung erfordert [33]. Entsprechend der frühen Phase der Implementierung sollten zukünftige Studien die Kenntnis entsprechender Angebote, digitale Kompetenzen und das Vertrauen in digitale, personalisierte Informationsangebote einbeziehen.

### Praktische Implikationen und Fazit

Durch den vorliegenden Beitrag werden Potenziale für die digitale Transformation der Informationsbereitstellung von Ärzt*innen für ihre Patient*innen aufgezeigt, die Patient*innen in Teilen von der eigenen Recherche und den damit einhergehenden Herausforderungen entlasten können. Während gute Voraussetzungen bestehen, dass Patient*innen nutzer*innenfreundliche und qualitativ hochwertige Angebote akzeptieren und nutzen, gilt es auf ärztlicher Seite, individuelle und systemische Barrieren abzubauen. Dazu kann vor allem eine Betonung des Nutzens digitaler Angebote beitragen. Es geht hier um die Zeitersparnis im Arbeitsalltag und eine bessere Wissensbasis informierter Patient*innen, die sich positiv auf ihr physisches und psychisches Wohlbefinden auswirken und auch den Anspruch an die Patient*innenzentrierung besser einlösen können. Allerdings braucht es dafür auch belastbare wissenschaftliche Erkenntnisse zum Einsatz solcher Angebote. Entsprechende Studien sollten Antworten auf die Fragen finden, welche Wirkung der Einsatz digitaler Informationsangebote auf die Beziehung zwischen Ärzt*innen und Patient*innen hat und wie sie die Genesung von Patient*innen beeinflussen. Zudem sollten Leitlinien die gesundheitsbezogene Wissensbasis der Bevölkerung sowie die Art der Informationsvermittlung weiter fokussieren, um Ärzt*innen diesbezüglich Orientierung zu bieten.
